# The scientific dichotomy and the question of evidence

**DOI:** 10.3402/qhw.v8i0.21846

**Published:** 2013-07-18

**Authors:** Karin Dahlberg

For some time now, the evidence debate has been very active. Methods are valued in relation to their power to guarantee evidence. A scientific dichotomy is present: the one between qualitative and quantitative approaches, between linguistic and numeric methods. Quantitative approaches building on numeric methods are held more scientific per se. Qualitative approaches building on linguistic methods are considered less scientific.

This dichotomy is not that new; it has existed for some hundred years. In short, the 17th-century revolution of natural sciences had an enormous impact on the development of all science and research. When the foundation for modern medicine was constructed, a mechanistic worldview was dominant and humans were understood as complex machines, ruled by the natural laws that regulate everything else in nature. Seen this way, health and—in particular—illness could be scientifically examined with methods that built upon mathematics and statistics. The picture was the same then as it is now: mathematics was understood as the cradle of science and that which can be examined numerically and described by statistics has higher scientific value.

The main problem of contemporary health science research is not the emphasis on numeric research and statistics; instead, it is the positivist isms that still dictate scientific research. These are atomism, reductionism and what I call categorism, and they illustrate the urge to divide complex wholes into groups of small and separate parts, preferably as least common denominators, which then can be weighed and measured.

Thereby, dichotomies and even dualisms have remarkable significance, such as body versus soul, health versus illness, objective versus subjective, sense versus sensibility, the rational versus the emotional. Illness is more compatible with this approach than with health, and as a consequence modern medicine deals more with illness than with health.

Furthermore, illness is being defined by diagnoses and symptoms that can be quantified and against which therapies can be evaluated. Diagnoses are even contra productive as they give no clues to understanding a person's health resources or how he/she can be supported in strengthening his/her health processes. Diagnoses are powerful categories, having the capacity to change a person's self-image and identity. Focus shifts from individual to groups, since statistics mean group orientation.

Diagnoses also have a normative effect—more and more of everyday existence is now being diagnosed. Not least alarming is it that modern medicine in such a way co-operates with the idea of the world as a market, where everything has a price.

In fact, even researchers who see the need for linguistic research approaches and who may actively argue against the dominance of numeric methods, themselves still work within the modern paradigm. They agree to the system of diagnoses, and by looking at the wide range of publications in international journals one can observe the main focus on illness. *International Journal of Qualitative Studies on Health and Well-Being* is one of the few exceptions.

Another example of the effects of the modern paradigm in the area of qualitative research is the high presence of research findings presented as “categories” or “themes,” but where it is not mentioned *of what* they are categories or themes. It is also easy to find numbers in qualitative research, such as the information of how many of the eight interviewees who reported an experience, information that has no relevance at all for the conclusions.

All methods, numeric as well as linguistic, have scientific value depending on what phenomenon is in focus and how the methods are employed. For the users of numeric methods, the main problem is the emphasis on the calculations and many decimals instead of paying enough attention to the character of the phenomenon in focus (how well it is aimed for numeric methods) and the interpretation of the results, for example, how to move from group values to individual and contextual values.

For the users of linguistic methods, it is also about choosing the most well-suited method, depending on the phenomenon and how it is best illuminated. If these researchers want their findings to make a difference in the practise of health care, they must carefully learn the ontology, epistemology and methodology of qualitative research, for example, how it is possible to understand the lived world of another human, how one can understand experiences that oneself has never been close to, how language as well as the lived body work in, for example, interviewing respectively in observation. These researchers need to know the difference between content and meaning in a narrative, and how it is epistemologically possible to understand the deep-lying meanings of the lifeworld.

Consequently, there is a fundamental need of the insights that have been developed by theory of science. However, this is a much more ambiguous demand than it may seem to be. Why? Because modern science got rid of all theory of science. With its emphasis on one and only “method” and the idea of “unified science,” there was no longer the need for questions of what the world is like, how humans are different from plants or stones, how one gets access to others’ experiences or how different methods work. When researchers in human or social sciences went about creating new methods with the means of language and words, they forgot to rebuild the theoretical ground on which the linguistic methods stand firm and which governs approaches that can stand up for evidence that is not numerically driven.

Phenomenology is the approach of in-between—it can never ever be reduced to empirism, psychologism, materialism, idealism, objectivism, subjectivism or constructivism, those that stem from the dualism of an outer and an inner world. In phenomenology, and the hermeneutics that spring from phenomenology, we find a wealth of theory of science that can inspire and enlighten researchers who want to advance in scientific research in the area of health and well-being and the care that strengthens health and well-being. The lifeworld theory can serve the purpose of understanding others’ lifeworlds, their intentionality and their meaning of the lived world. Phenomenology also has formed reflections upon how to go about not letting one's own pre-suppositions making judgments, formulating the findings.

Grounded theory is another approach that may serve a new generation of researchers. Its founders state that they were inspired by phenomenology, which may be a reason why this approach contributes to many more interesting results than most vague “qualitative approaches.” The interest of Grounded theory in generating “grounded theory” is one of the advantages of the approach. This is a claim that all researchers should address.

Even if one does not go all along that road, which may demand a synthesis of more than one study, researchers can reflect upon the idea of relating meanings in the data to each others or relating their findings to existing theory or others’ findings, in order to generate more advanced findings that can inform clinical practitioners.

In such a manner, approaches of qualitative research will make use of their own label: high-quality research that can inform the health care practitioners on how they can help people support and strengthen their health processes, their experience of vitality and a good life rhythm, and thereby ensure that these people find a meaningful way to realise their life projects.

*Karin Dahlberg* Professor in Caring Science

